# Experimental Validation of a Compound Control Scheme for a Two-Axis Inertially Stabilized Platform with Multi-Sensors in an Unmanned Helicopter-Based Airborne Power Line Inspection System

**DOI:** 10.3390/s16030366

**Published:** 2016-03-11

**Authors:** Xiangyang Zhou, Yuan Jia, Qiang Zhao, Ruixia Yu

**Affiliations:** 1 School of Instrumentation Science and Opto-electronics Engineering, Beihang University (BUAA), Beijing 100191, China; jiayuan@buaa.edu.cn; 2 Shanghai Institute of Satellite Engineering, Shanghai 200240, China; zhaoqiangbuaa@163.com; 3 School of Mechanical Engineering, University of Science and Technology Beijing, Beijing 100083, China; yrx_nx@163.com

**Keywords:** unmanned helicopter, airborne power line inspection, two-axis inertially stabilized platform, voltage fluctuation, back electromotive force

## Abstract

A compound control scheme is proposed to achieve high control performance for a two-axis inertially stabilized platform (ISP) with multi-sensors applied to an unmanned helicopter (UH)-based airborne power line inspection (APLI) system. Compared with the traditional two closed-loop control scheme that is composed of a high-bandwidth rate loop and a lower bandwidth position loop, a new current loop inside rate loop is particularly designed to suppress the influences of voltage fluctuation from power supply and motor back electromotive force (BEMF) on control precision. In this way, the stabilization accuracy of the ISP is greatly improved. The rate loop, which is the middle one, is used to improve sensor’s stability precision through compensating for various disturbances. To ensure the pointing accuracy of the line of sight (LOS) of multi-sensors, the position loop is designed to be the outer one and acts as the main feedback path, by which the accurate pointing angular position is achieved. To validate the scheme, a series of experiments were carried out. The results show that the proposed compound control scheme can achieve reliable control precision and satisfy the requirements of real APLI tasks.

## 1. Introduction

The electric power distribution system is an important part of electrical power systems for delivering electricity to consumers [1]. Preventive and breakdown maintenance of the power grid are typically a costly legal public safety responsibility in most countries [2]. In order to detect defects as early as possible and to efficiently plan the required maintenance activities, distribution networks are regularly inspected [3]. Power line inspection is a vital function for electricity supply [4]. So far, the inspection of power line corridors is mainly carried out visually or by helicopters. However, visual inspection is very labor-intensive and inefficient [5]. In comparison to the foot patrol inspection, inspection by helicopter is more efficient [6]. In recent years, due to their potential applications in different fields, great interest in the utilization of unmanned aerial vehicles (UAVs) has arisen [7]. As a new method, UAV-based power line inspection utilizes modern flight control techniques, image photography and recognition techniques to carry out fast inspections from high altitude and over far distances [8]. Therefore, unmanned helicopter (UH)-based power line inspection is becoming very attractive for it is more reliable, effective and achieves detection of more faults [2,3,9].

In an airborne power line inspection (APLI) system, an unmanned helicopter (UH) carrying imaging sensors such as a CCD camera and an infrared scanner moves along the power line. The conditions of the power line can then be estimated based on the obtained images [10]. An APLI system generally consists of an autonomous helicopter, an inertially stabilized platform (ISP), a position and orientation system (POS) and multiple sensors, such as a visible light camera, infrared camera, ultraviolet camera, laser scanner, and so on. The system can intelligently detect the power transmission line faults. All multi-sensors are installed in the ISP, including the inertial measurement unit (IMU) of POS, so the field vision of the multiple sensors can be regulated to the required direction and target with the output of POS [11].

A two-axis ISP with high control performance, which is used to support and stabilize the multiple sensors so that the sensors’ line of sight (LOS) can track the APLI target accurately in real time, is indispensable for an APLI system [7]. The control task for such an ISP is to keep the LOS (optical axis) of the optical or optoelectronic payload still, even in the presence of some unwanted rotational motion of the carrier [12]. Besides, with the help of a two-axis ISP, the sensors’ LOS can track the lines and remain steady all along by rejecting various disturbing torques, such as vibration, air turbulence, wind gusts and so on. Therefore, there is much interest among researchers to investigate control methods with higher accuracy and stability by rejecting various disturbances [13]. In [12], a feedforward scheme for a two-axis ISP to reject a periodic disturbing torque acting on the payload due to static mass unbalance is reported. In [14], an adaptive backstepping control method based on the LuGre model is put forward to decrease the influences of friction on control precision of a three-axis ISP. In [15], the extended Kalman Filter (EKF) is used to compensate friction disturbance for a second order system with low-pass PD controller. In [16], a robust adaptive output feedback controller is applied to a cart-crane system. In [17], an acceleration–based feedforward approach is proposed to realize imbalance torque compensation of a three-axis ISP. In [18], a model-based feedforward compensation approach is applied to a rotary table system. In [19], an anti-disturbance compensation control algorithm based on adaptive robust control idea is applied for a three-axis swing turntable system. In addition, due to the existing nonlinearity and time-varying uncertainty in the practical engineering application, improved PID controller is usually adopted to compose a complex advanced PID control method [20].

In order to achieve the necessary control performance required by an APLI system, a two-axis ISP with both of high control precision and stability is crucial in an inspection task, meaning a comprehensive control scheme of ISP should be successfully designed. Typically, the control system of an ISP is configured as a high-bandwidth rate loop inside a lower bandwidth pointing or tracking position loop [21]. The ISP might be viewed as a means for removing high-frequency disturbances and controlling the LOS, whereas the pointing and tracking loops have the task of removing the lower frequency parallactic motion and perhaps any bias or drift in the ISP rate loop [22]. Therefore, a dual closed-loop PID control scheme is widely used as a main method for the control system design of multi-axis ISP that was surveyed in [21,22]. However, in the dual closed-loop scheme, the disturbances caused by voltage fluctuation from power supply and motor back electromotive force (BEMF) are not compensated by an effective closed-loop, which can influence the stabilization accuracy of ISP. Since the motor armature current will keep following the change of the voltage fluctuation, thus, the control precision and stability of ISP will decrease. Therefore, to improve the dynamic performance of the system, the motor armature current should be kept stable in the design of control system to ensure the linearity of control torque. It has been proved that suppressing the torque ripple from the motor drive of a servo system can significantly improve system performance by reducing speed fluctuations. Therefore, in the design of control scheme of two-axis ISP, the disturbances of motor unstable armature current caused by voltage fluctuation and BEMF should be compensated on the basis of dual closed-loop scheme.

In this paper, to reduce the influences of voltage fluctuation of power supply and motor BEMF on the stabilization accuracy of a two-axis ISP, on the basis of dual closed-loop PID control scheme, a specialized current feedback closed-loop is introduced to improve the dynamic and static performance of rate loop. Together with original dual closed-loops, a compound control scheme is configured. Through this scheme, the linearity of control torque is improved. To verify the methods, serial experiments are carried out to a two-axis ISP for APLI applications.

## 2. Background Analysis

### 2.1. APLI System

**Figure 1:**
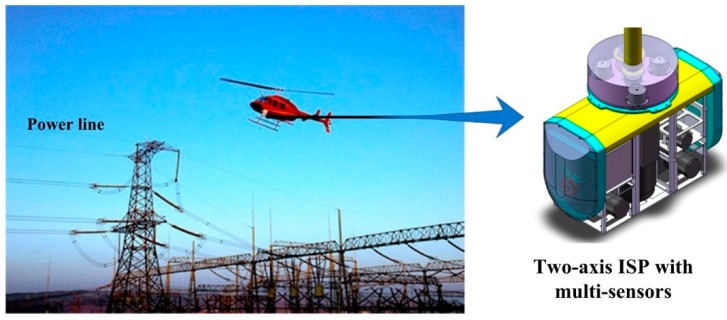
Schematic diagram of an APLI system and the two-axis ISP with multi-sensors.

Figure 1 shows an APLI system and a two-axis ISP with multiple sensors. An unmanned helicopter (UH) is usually chosen as a mobile aviation aircraft which can take off and land freely in a complex geographic environment. Imaging sensors such as a CCD camera, infrared scanner and ultraviolet scanner are carried by the ISP, which is mounted between the sensors and the aviation platform. The sensors and ISP are assembled as a pod system hung below the helicopter. The pod system can communicate with a supervisory computer. The control system of ISP can get information from the supervisory computer and other inertial sensors to realize stabilizing function for the LOS of camera [6].

### 2.2. Structure of Two-Axis ISP System

**Figure 2:**
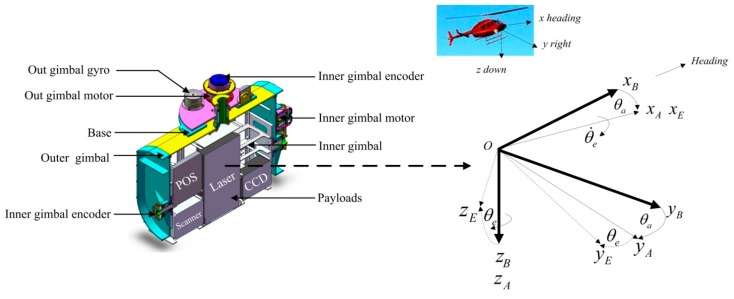
The structural diagram of a two-axis ISP with multi-sensors in an APLI system.

As shown in Figure 2, for a two-axis ISP system, the inner gimbal allows elevation of the payload, and the outer gimbal allows a rotation in azimuth angle. The payload consists of two CCD cameras, an infrared scanner, an ultraviolet scanner, and a laser scanner. Torque motors and gear linkages are used to drive two gimbals’ rotation. Two fiber-optic gyroscopes, one POS and two encoders are attached to the payload to measure its rotation angles, rates and accelerations [10].

The POS, which is mainly composed of three main components, *i.e.*, inertial measurement unit (IMU), GPS receiving antenna and data processing system, is used to provide an accurate reference of position and attitude in inertial space for control system of ISP and imaging sensors through measuring imaging sensor’s angular movement.

### 2.3. Working Principle of Two-Axis ISP System

**Figure 3:**
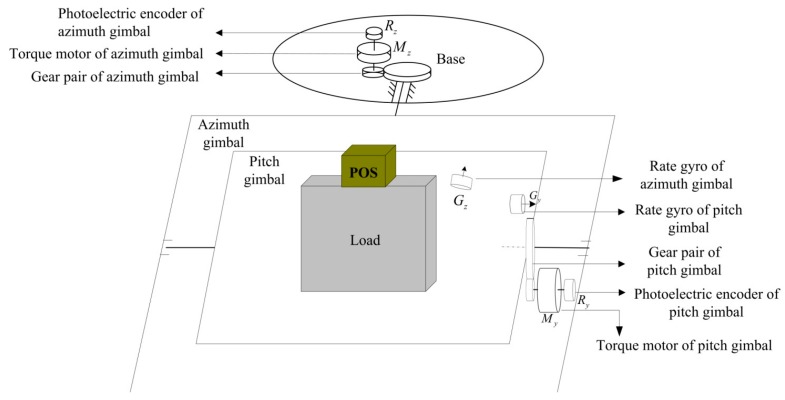
The structure diagram of two-axis ISP control system in APLI system.

Figure 3 shows the structure diagram of a two-axis ISP control system in the APLI system. We can see that the ISP consists of two gimbals, which are an azimuth gimbal (A-gimbal) and a pitch gimbal (P-gimbal) [7,13]. The P-gimbal is assembled on the A-gimbal and can rotate around the Y_p_ axis. The A-gimbal is assembled on the base of the aviation platform and can rotate around the Z_a_ axis. G_p_ and G_a_ stand for the rate gyros that measure the inertial angular rate of the P-gimbal and A-gimbal, respectively. E_p_ and E_a_ respectively stand for the photoelectric encoders installed on the P-gimbal and A-gimbal, which are used for measuring the relative angles between gimbals. M_z_ and M_y_ respectively stand for the gimbal servo motors which drive the A-gimbal and P-gimbal to keep them steady in inertial space. Ay represents the accelerometer installed on the P-gimbal that is used to measure the gimbal’s rotary angular acceleration.

## 3. Control Scheme of Two-Axis ISP

### 3.1. The Traditional Dual Closed-Loop Control Structure

**Figure 4:**
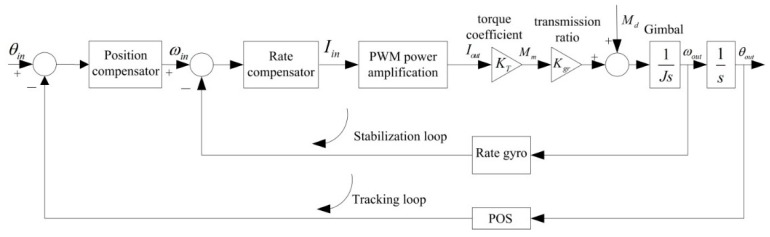
Schematic diagram of dual closed-loop PID control structure.

Figure 4 shows the schematic diagram of a traditional dual closed-loop control structure. We can see that this structure is composed of the inner rate loop and outer position loop. The inner rate loop uses a rate gyro to measure the angular rate of the gimbal in the inertial space, which is used to compensate the difference between the rate command input and the angular rate of the gimbal, eventually improving the steady-state precision. As the main feedback path, the outer position loop takes the attitude angle measured by POS as accurate reference to ensure the accurate pointing of the LOS. However, since there is no current loop inside, the influences of voltage fluctuation and the motor BEMF cannot be suppressed, eventually leading to decreased control precision and destabilization of the ISP.

### 3.2. The Three Closed-Loop Compound Control Structure

**Figure 5:**
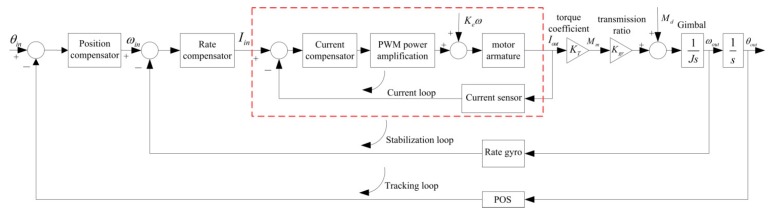
Schematic diagram of three closed-loop compound control structure.

Figure 5 shows the control structure of the proposed three closed-loop compound control scheme. On the basis of dual closed-loop PID control scheme, a specialized current feedback closed-loop is designed to improve the dynamic and static performance of rate loop, which is highlighted in a red dotted box in Figure 5. Thus, the control system structure is composed from the inside to the outside of a current loop, rate loop and position loop, respectively. By adding the current loop, the disadvantages of a dual closed-loop PID control structure are overcome. As a result, the control precision and stabilization of ISP are improved since the influences of voltage fluctuation of power supply and the motor EMF are suppressed by the added current loop inside.

**Figure 6:**
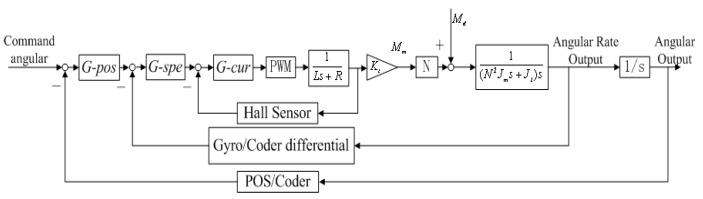
Block diagram of three-loop compound PID control structure of two-axis ISP control system in APLI system.

Figure 6 shows the block diagram of the proposed three closed-loop compound control structure. The blocks of *G-pos*, *G-spe* and *G-cur* separately represent the controllers in the position loop, rate loop and current loop; the PWM block represents the power amplification used for amplifying the current to drive the torque motor; the symbol *L* represents the inductance of a torque motor and *R* represents the resistance; the symbol K*_t_* represents the torque coefficient of the motor and *N* is the transition ratio from the torque motor to the gimbals; *J_m_* represents the moment of inertia of the motor and *J_l_* represents the moment of inertia of the gimbals along the rotation axis.

For the structure of the three closed-loop compound control, the system bandwidths of each loop increase from the outer loop to the inner loop, meaning the current loop has the highest bandwidth among the three closed-loops, leading to the rapidest response speed. As for each gimbal control system, the function of rate loop, *i.e.*, stabilization loop, is to minimize the effects of external disturbances, in which a rate gyro is used as feedback sensor to directly measure inertial LOS rate. Therefore, the compensated torques for disturbance rejection are determined by the stabilization controller in response to the combined command input and LOS inertial rate feedback [21]. The function of position loop is to keep tracking the position objective in real time, in which a POS is used as the position sensor.

#### 3.2.1. Design of the Current Loop Controller

**Figure 7:**
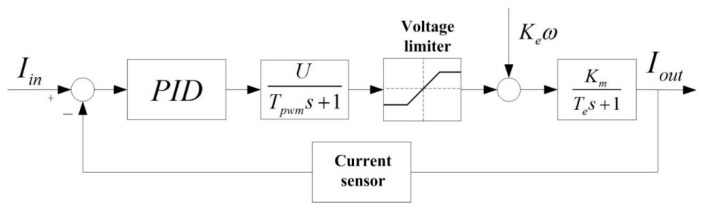
The structural diagram of current loop.

Figure 7 shows the structural diagram of the current loop for a pitch system. A PID controller is adopted in the design. We can see that the drive model of the motor power is composed of the motor model, the current detection model, the current controller model and PWM power drive model. In Figure 7, *I_in_* and *I_out_* stand for the input current and output current, respectively. *U*/*T_pwn_s*+1 is the transfer function of the PWM power drive unit, in which *U* is the power supply voltage and *T_pwm_* is the PWM switching period. *K_e_ω* stands for the motor counter electromotive force, in which *ω* and *K_e_* are the motor speed and BEMF coefficient, respectively. *K_m_*/*T_e_s*+1 is the transfer function of the motor armature, in which *T_e_* and *K_m_* stand for the time constant of the armature magnetic and armature current coefficient, respectively.

In order to get a better response speed and stability precision, the current loop controller is designed with a PI serial correction. Assume that the current controller is *K_I_*(*τ_I_s*+1/s), where *τ_I_* is set to equal the time constant of the magnetic armature *T_e_* in order to offset the electromagnetic inertia of motor at a greater extent. Thus, the transfer function of the open current loop can be obtained as:

(1) $$\begin{array}{l} G_{opc}=\frac{K_{I}\cdot K_{m}\cdot U}{(T_{pwm}s+1)s}=\frac{K_{I}\cdot K_{m}\cdot U}{T_{pwm}}\cdot \frac{1}{s^{2}+\frac{1}{T_{pwm}}s}\\ \left\{ \begin{array}{l} 2\xi \omega_{n}=\frac{1}{T_{pwm}}\\ \omega_{n}^{2}=\frac{K_{I}\cdot K_{m}\cdot U}{T_{pwm}} \end{array}\right. \end{array}$$

**Figure 8:**
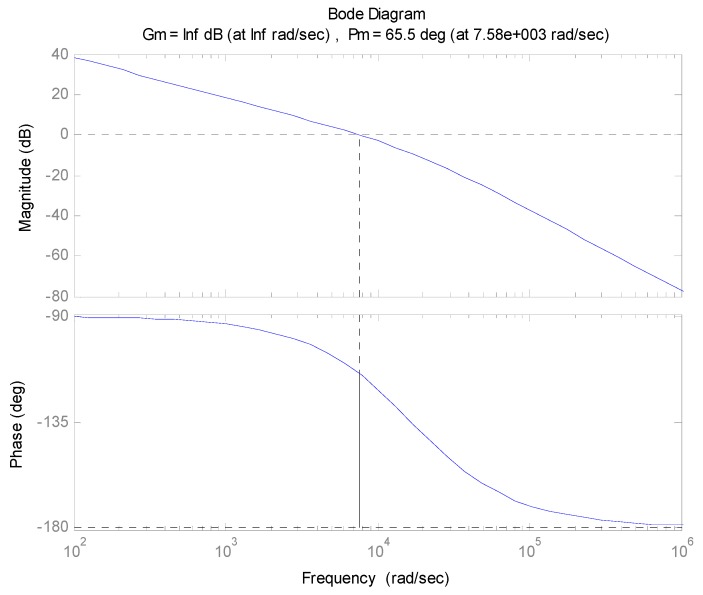
Simulation correction results of open current loop for pitch system.

We can see that the current loop system is a second-order system. When the damping ratio is *ξ* = 0.707, system will be an optimized second-order system, so if *ξ* is substituted into Equation (1), the *ω_n_* and PID parameter *K_I_* are then obtained. Figure 8 shows the Bode diagram of the pitch gimbal’s open current loop. We can see that the phase margin and bandwidth are 65.5 degrees and 7580 rad/s, respectively.

#### 3.2.2. Design of the Rate Loop Controller

**Figure 9:**
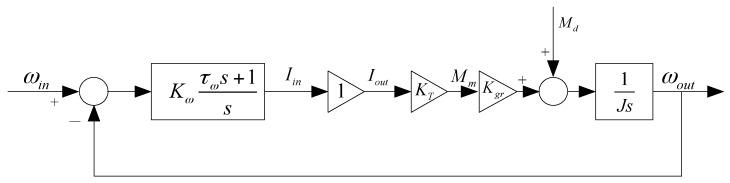
The structural diagram of the rate loop.

Figure 9 shows the structural diagram of rate loop. Since the bandwidth of the current loop is much greater than that of the rate loop, the current loop can be approximately taken as a proportional element whose coefficient is equal to 1. Thus, the structure of the rate loop is simplified, which is helpful for the analysis and correction of the rate loop. The PI series controller is adopted in the design, which is represented as *K_ω_*(*τ_ω_s*+1/*s*). Besides, *K_T_* and *K_gr_* stand for the coefficient of motor torque and transmission ratio of gear linkage, respectively.The transfer function of rate gyro is 1/*T_G_s*+1, *M_d_* stands for the disturbance torques, *J* stands for the rotational inertia of the gimbal together with the payload. The time constant *T_G_* is small so it can be neglected.

The open loop transfer function of the rate loop is as follows:

(2) $$G_{op\omega }=K_{\omega }\cdot K_{T}\cdot K_{gr}\cdot \frac{\tau_{\omega }s+1}{Js^{2}}$$

The frequency characteristic is:

(3) $$G_{op\omega }(j\omega )=K_{\omega }\cdot K_{T}\cdot K_{gr}\cdot \frac{\tau_{\omega }\cdot j\omega +1}{J\cdot j\omega \cdot j\omega }$$

Since the mechanical resonance frequency of the ISP, *ω_R_*, can be obtained by modal analysis and testing, the open loop cut-off frequency of the rate loop, *ω_c∙ω_*, can be set to be 1/5 of *ω_R_* and then obtained as : *ω_c∙ω_ =* 6.28 rad/s. In order to make system have better performance, such as stability, short regulation time, small overshoot, and so on, the phase margin of the open rate loop is designed to be 70 degrees. It means that when *ω_c∙ω_ =* 6.28 rad/s, there is ∠j∙9*τ_ω_* + 1 = 70°. Thus, we get: *τ_ω_* = 1∙*tg*70°(1/6.28) = 0.4375.

When *ω_c∙ω_ =* 6.28 rad/s, ${\left|G_{op\omega }\right|}_{\left(j\omega_{c\cdot \omega }\right)}=1$. From Equation (3), we get:

(4) $$K_{\omega }\cdot K_{T}\cdot K_{gr}\cdot \frac{\sqrt{{\left(\tau_{\omega }\omega_{c\cdot \omega }\right)}^{2}+1}}{J\cdot \omega_{c\cdot \omega }\cdot \omega_{c\cdot \omega }}=1$$

If *ω_c∙ω_ =* 6.28 rad/s and *τ_ω_ =* 0.4375 are substituted into Equation (4), then *K_ω_* is obtained.

#### 3.2.3. Design of the Position Loop Controller

**Figure 10:**
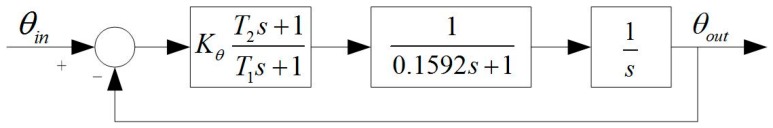
The structural diagram of position loop.

Figure 10 shows the structural diagram of position loop. The PI series controller is adopted in the design, which is represented as *K_θ_*(*T*_2s_ + 1/*T*_1_s + 1). The open loop cut-off frequency of the position loop is set to be 1/4 of *ω_c∙ω_*, *i.e.*, *ω_c∙θ_* = 1.57 rad/s. As mentioned above, the open loop cut-off frequency of rate loop, *ω_c·ω_*, is equal to 6.28 rad/s. Since the rate loop is as an inner loop inside the tracking loop, it can be taken as a first order inertial element whose time coefficient is *T_ω_* = 1/*ω_c·ω_* = 0.1592 s.

So the approximately equivalent closed-loop transfer function of rate loop is as follows:

(5) $$\phi_{\omega }=\frac{1}{T_{\omega }s+1}$$

Then the transfer function of open position loop is approximately expressed as follows:

(6) $$G_{op\theta }=K_{\theta }\frac{T_{2}s+1}{T_{1}s+1}\cdot \frac{1}{T_{\omega }s+1}\cdot \frac{1}{s}$$

We set 1/*T_2_* = 0.1*ω_c·θ_* and *T_1_* = 3*T_2_*. Since the cut-off frequency of open position loop is designed as *ω_c·θ_* = 1.57 rad/s, *T_1_* and *T_2_* are obtained. Thus, two known constants *T_1_* and *T_2_* are substituted into ${\left|G_{op\theta }\right|}_{\left(j\omega_{c\cdot \theta }\right)}$ = 1, then the PID parameter *K_θ_* is obtained.

After calculation, the bandwidth and phase margin of the pitch gimbal’s open position loop are 1.57 rad/s and 72.2 degree, respectively. The bandwidth of the pitch gimbal’s closed loop is 2.18 rad/s, which meets the requirement of tracking POS.

## 4. Experiments and Results

### 4.1. Testing under Static Base Conditions

**Figure 11:**
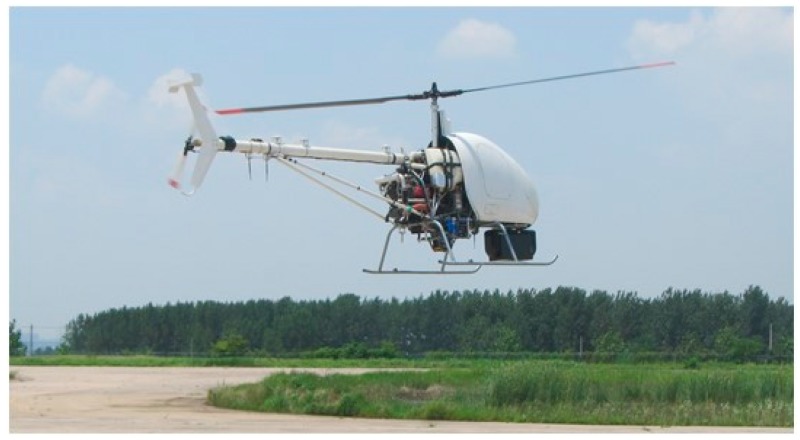
The experimental UH-based APLI system.

A series of experiments are carried out to a two-axis ISP developed for an UH-based APLI system to verify the proposed scheme. Figure 11 shows the experimental UH-based APLI system. The flight time of each inspection is about 0.5–1 h.

**Figure 12:**
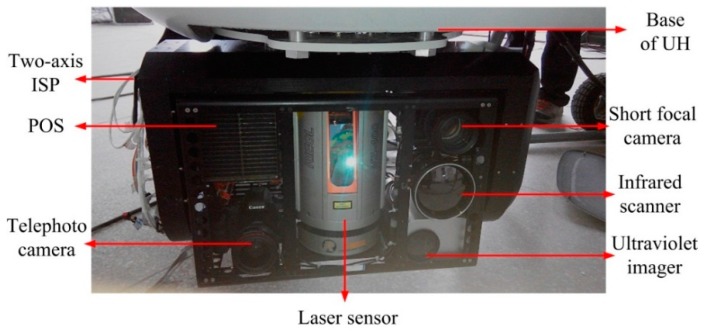
A picture of the two-axis ISP with multiple sensors under the static base testing conditions.

Figure 12 shows the picture of the two-axis ISP with multiple sensors under the static base testing conditions. In the static testing, the two-axis ISP is fixed under the bottom of the UH and tests are conducted in a stable environment. The main physical parameters of the ISP are as follows: maximum load capacity is 36 kg and the weight of ISP is 20 kg, respectively. The range of azimuth rotation angle is 0°–360°, the maximum pitch rotation angle range is ±50°.

#### 4.1.1. Tracking Performances

**Figure 13:**
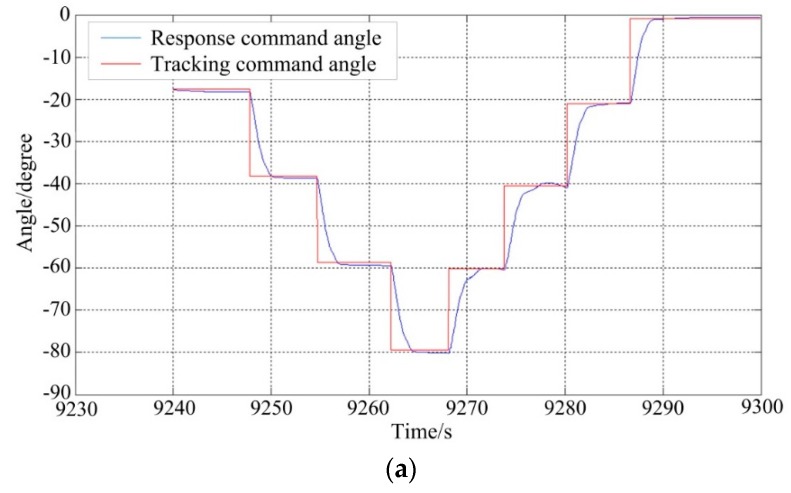

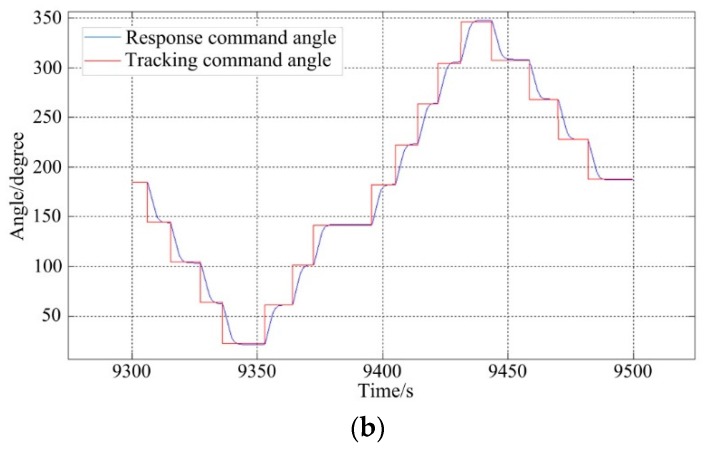
The tracking results of angle position under static testing: (**a**) the pitch system; (**b**) the azimuth system.

Figure 13 shows the tracking results of ISP under static base testing conditions. It can be seen that the ISP can steadily track the command instructions with high accuracy. For the pitch system, the command angles are from −20° to −80° and then reverse to 0° with a short stabilization time per 20° interval.

For the azimuth system, the stabilization performances is tested per 40° interval from 180° to 20° and then reverse to 320°. The maximum overshoot is less than 1.5° for both gimbal systems.

#### 4.1.2. Stabilization Performance

(1) Pitch System

**Figure 14:**
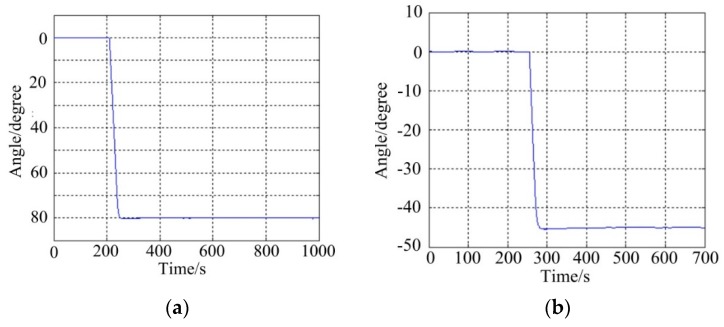
Stabilization results of the pitch system under static base conditions: (**a**) instruction command angle from 0° to −80°; (**b**) instruction command angle from 0° to −45°.

Figure 14 shows the stabilization results of the pitch system under static base testing conditions, which include two cases: (a) represents a command angle from 0° to −80° and (b) represents a command angle from 0° to −45°. It can be seen that the angular position errors of the pitch system under tracking −80° and −45° are less than 0.0165° (RMS) and 0.0155° (RMS), respectively, meaning that the compound control scheme can achieve a high static stabilization precision.

(2) Azimuth System

**Figure 15:**
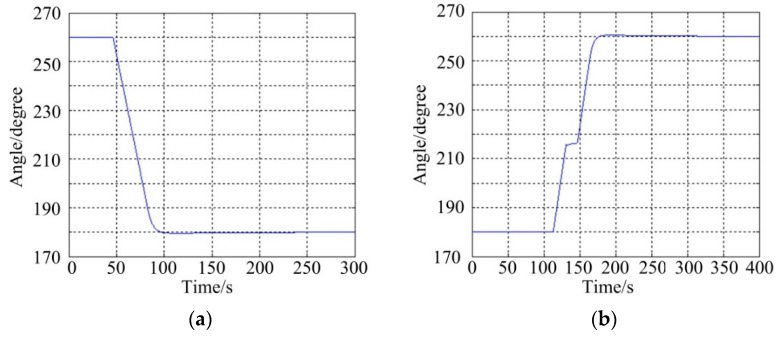
Stabilization results of the azimuth system under static base conditions: (**a**) command angle from 260° to 180°; (**b**) command angle from 180° to 260°.

Figure 15 shows the stabilization results of the azimuth system under static base testing conditions that also include two cases: (a) represents a command angle from 260° to 180° and (b) represents a command angle from 180° to 260°. It can be seen that the angular position errors of azimuth system when tracking 180° and 260° are less than 0.0102° (RMS) and 0.0125° (RMS), respectively. Likewise, this means the compound control scheme of the azimuth system can achieve a reliable static stabilization performance.

### 4.2. Testing under Dynamic Base Conditions

**Figure 16:**
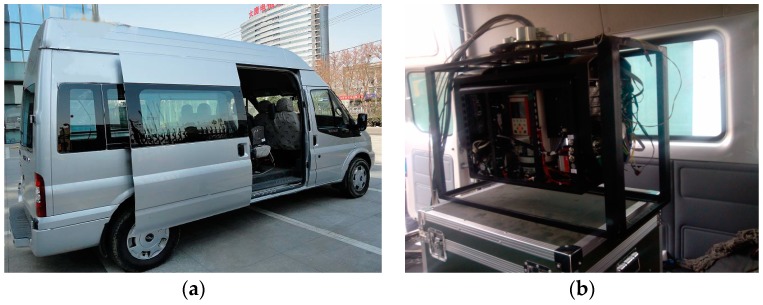
Pictures of the experimental equipment in dynamic experiments: (**a**) moving vehicle; (**b**) two-axis ISP inside the vehicle.

As shown in Figure 16, to further validate the dynamic performance of the proposed scheme, dynamic experiments were carried out on a movable vehicle. In experiments, the ISP is fixed on the top of a box through a transitional support frame.

**Figure 17:**
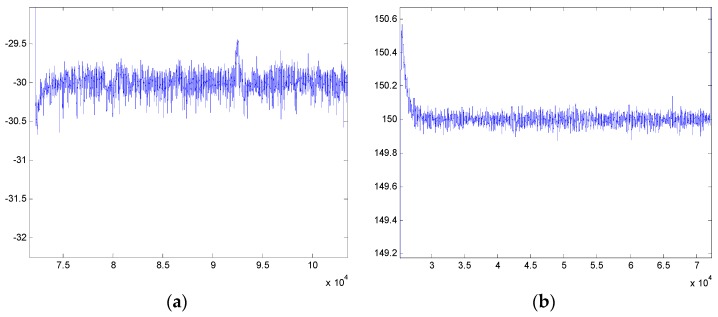
Stabilization results in dynamic experiments: (**a**) pitch system; (**b**) azimuth system.

Figure 17 shows the testing results in the real movable vehicle experiments. It can be seen that the stabilization errors of the pitch system and azimuth system are less than 0.5° and 0.2° in case of tracking the angular positions of −30° and 150°, respectively, meaning a reliable dynamic performance that can reject effectively random disturbances in a moving base scenario.

### 4.3. Testing the UH-Based APLI System in the Air

**Figure 18:**
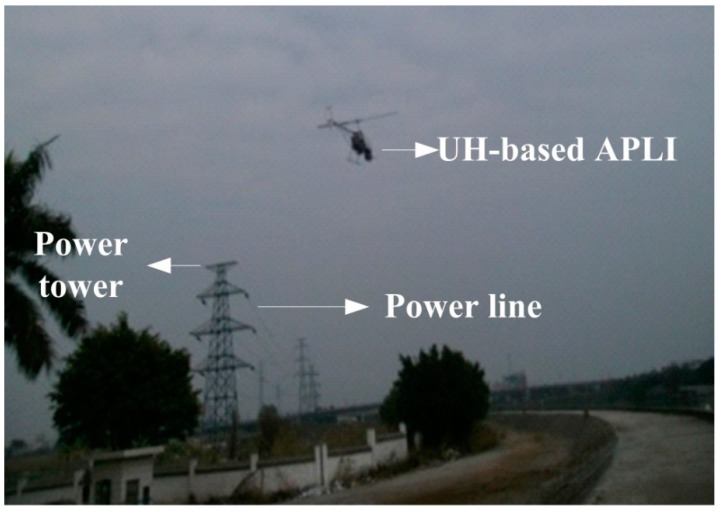
The picture of the experiments for a real UH -based APLI system.

In order to verify the effectiveness of the control system, a two-axis ISP with the proposed compound control scheme was applied in a real UH-based APLI system to detect the defects of a high- voltage power line in Foshan, Guangdong, China, as shown in Figure 18.

**Figure 19:**
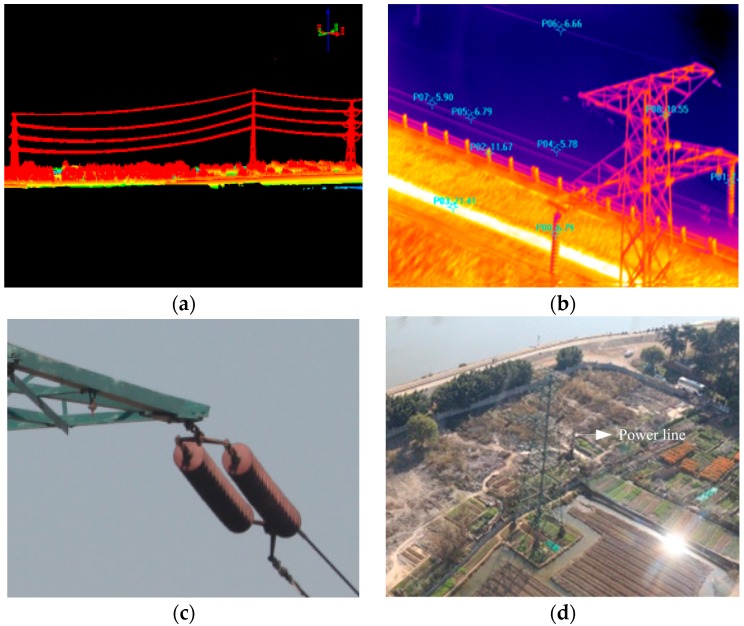
The partial real imaging data acquired by the APLI system using: (**a**) laser scanner; (**b**) infrared scanner; (**c**) CCD short focal distance camera; (**d**) telephoto camera.

Figure 19 shows the partial imaging data acquired by the APLI system. It can be seen the imaging data are clear regardless on which of the multiple sensors is used, meaning that the control performance of the two-axis ISP is reliable, and can satisfy the imaging requirements of real APLI system.

**Figure 20:**
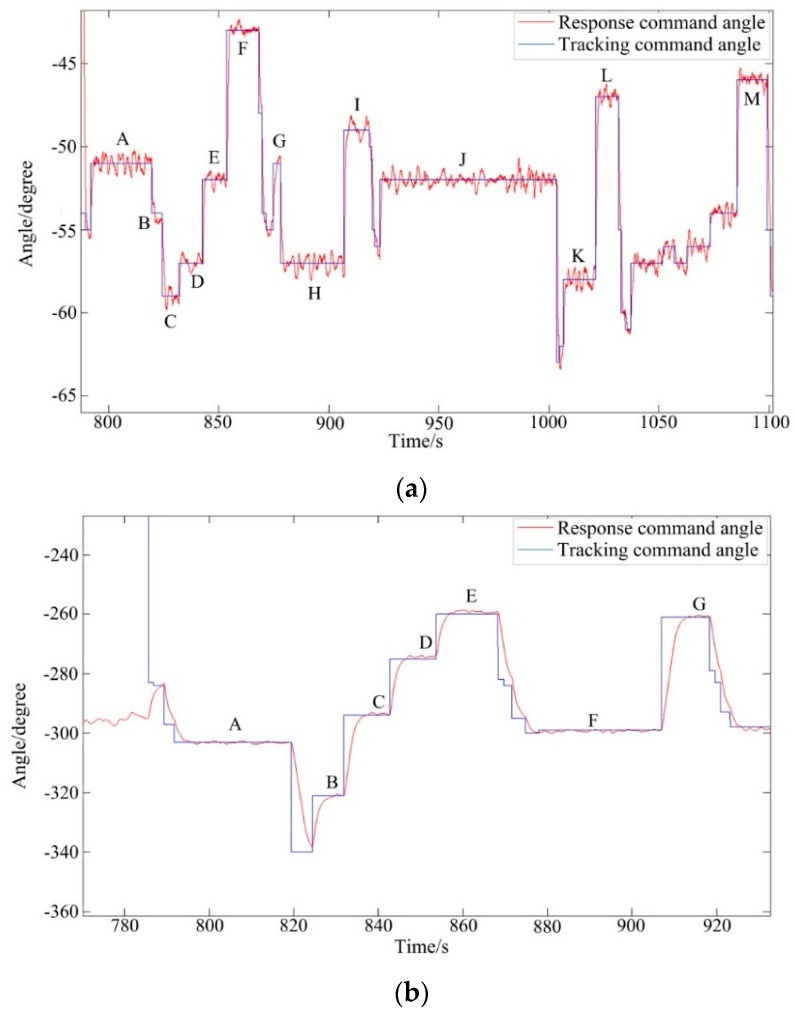
The tracking results of angle position of the real UH-based APLI system in air: (**a**) the pitch system; (**b**) the azimuth system.

Figure 20 shows the experimental results of the two-axis ISP of the real UH-based APLI system in the air. It can be seen that under real flight conditions, the ISP still has high tracking accuracy with the command instructions, similar to the results in the movable vehicle experiments. The steady-state tracking errors of the pitch system and azimuth are 0.3456° (RMS) and 0.3481° (RMS), respectively. The RMS values of different trails in Figure 20a,b are summarized in Table 1 and Table 2, respectively.

## 5. Conclusions

In this paper, to improve the stabilization accuracy of a two-axis ISP used in an UH-based APLI system, a compound control scheme is proposed. To reduce the influences of power supply voltage fluctuations and motor BEMF, a specialized current feedback closed-loop is introduced to improve the dynamic and static performance of the rate loop on the basis of a dual closed-loop PID control scheme. The control performances of the scheme are analyzed and simulated. Particularly, a series of experiments are carried out to validate the scheme. The experimental results under real flight conditions show that the ISP has high tracking accuracy with the command instructions, whose steady-state tracking errors of the pitch system and azimuth are 0.3456° (RMS) and 0.3481° (RMS), respectively. By using a two-axis ISP operated with the method, reliable imaging data of multiple sensors are acquired for a real APLI system. The results validate that the proposed compound control scheme effectively improves the stabilization accuracy of a two-axis ISP.

## Figures and Tables

**Table 1:** Steady state RMS errors of each tracking angle for pitch gimbal.

Number	Tracking Angles (Degree)	RMS	Number	Tracking Angles (Degree)	RMS
A	−51	0.3982	H	−57	0.4200
B	−54	0.4642	I	−49	0.4330
C	−59	0.3773	J	−52	0.3413
D	−57	0.3563	K	−58	0.4379
E	−52	0.3088	L	−47	0.2987
F	−43	0.1925	M	−46	0.2843
G	−51	0.2819			

**Table 2:** Steady state RMS errors of each tracking angle for azimuth gimbal.

Number	Tracking Angles (Degree)	RMS	Number	Tracking Angles (Degree)	RMS
A	−303	0.3164	E	−260	0.4105
B	−321	0.2937	F	−299	0.3997
C	−294	0.3743	G	−261	0.3162
D	−275	0.3832			
